# Molecular Regulation of Toll-like Receptors in Asthma and COPD

**DOI:** 10.3389/fphys.2015.00312

**Published:** 2015-11-09

**Authors:** Li Zuo, Kurt Lucas, Christopher A. Fortuna, Chia-Chen Chuang, Thomas M. Best

**Affiliations:** ^1^Radiologic Sciences and Respiratory Therapy Division, The Ohio State University Wexner Medical Center, School of Health and Rehabilitation Sciences, The Ohio State University College of Medicine, Columbus, Ohio State UniversityColumbus, OH, USA; ^2^Interdisciplinary Biophysics Graduate Program, The Ohio State UniversityColumbus, OH, USA; ^3^Multiphase Chemistry Department, Max Planck Institute for ChemistryMainz, Germany; ^4^Division of Sports Medicine, Department of Family Medicine, Sports Health and Performance Institute, The Ohio State University Wexner Medical CenterColumbus, OH, USA

**Keywords:** antioxidant, DAMP, PAMP, polymorphism, reactive oxygen species, TLR

## Abstract

Asthma and chronic obstructive pulmonary disease (COPD) have both been historically associated with significant morbidity and financial burden. These diseases can be induced by several exogenous factors, such as pathogen-associated molecular patterns (PAMPs) (e.g., allergens and microbes). Endogenous factors, including reactive oxygen species, and damage-associated molecular patterns (DAMPs) recognized by toll-like receptors (TLRs), can also result in airway inflammation. Asthma is characterized by the dominant presence of eosinophils, mast cells, and clusters of differentiation (CD)4^+^ T cells in the airways, while COPD typically results in the excessive formation of neutrophils, macrophages, and CD8^+^ T cells in the airways. In both asthma and COPD, in the respiratory tract, TLRs are the primary proteins of interest associated with the innate and adaptive immune responses; hence, multiple treatment options targeting TLRs are being explored in an effort to reduce the severity of the symptoms of these disorders. TLR-mediated pathways for both COPD and asthma have their similarities and differences with regards to cell types and the pro-inflammatory cytotoxins present in the airway. Because of the complex TLR cascade, a variety of treatments have been used to minimize airway hypersensitivity and promote bronchodilation. Although unsuccessful at completely alleviating COPD and severe asthmatic symptoms, new studies are focused on possible targets within the TLR cascade to ameliorate airway inflammation.

## Introduction

Asthma and chronic obstructive pulmonary disease (COPD) are two respiratory diseases characterized by an accumulation of inflammatory cells in the respiratory tract, which leads to subsequent airflow obstruction (Singh and Busse, 2006; Quint and Wedzicha, 2007). It is estimated that 25 million people in the United States alone are affected by asthma (Centers of Disease Control and Prevention, 2013), and approximately 20 million people are diagnosed with COPD (American Lung Association). Ranking as the third leading cause of death in the United States, COPD shares similar symptomatic features with asthma. However, the mechanisms underlying the pathophysiology of these two diseases are distinct (Barnes, 2008). For instance, the instigators of airway inflammation are different for COPD (neutrophils) and asthma (eosinophils) (Barnes, 2000). Most notably, patients with COPD experience progressive and irreversible structural alterations, including airway remodeling and alveolar destruction, which ultimately result in breathing difficulties; whereas airway obstruction is often reversible in asthma (Barnes, 2008; Baines et al., 2011). Periodic exacerbations of COPD can worsen respiratory symptoms and markedly increase the risk of mortality (Domej et al., 2014).

Airway inflammation plays an essential role in the development of both asthma and COPD. Undoubtedly, variations in the inflammatory process between the two diseases underlie the unique immunopathologies of asthma and COPD. Emerging evidence suggests that toll-like receptors (TLRs) may be associated with the aberrant stimulation of immune responses, possibly contributing to the chronic inflammation seen in asthma (Phipps et al., 2007). TLRs are a subgroup of pattern recognition receptors (PRRs), which are antigen-sensitive, responsible for innate immunity (Kawai and Akira, 2010). Within the TLR family, there are 10 members that are active in humans (Warren, 2005). Generally, most of these TLRs have been split into two sub-groups. TLR1, TLR2, TLR4, TLR5, TLR6, and TLR11 comprise the first group and are primarily expressed on the cell surface; the function of this group is to recognize the components of microbial membranes (Blasius and Beutler, 2010; Kawai and Akira, 2010). The second sub-group is composed of TLR3, TLR7, TLR8, and TLR9. These TLRs are expressed intracellularly in vesicles (e.g., lysosomes, endosomes, and the endoplasmic reticulum) and can target microbial nucleic acids (Blasius and Beutler, 2010; Kawai and Akira, 2010). Serving as the first line of host defense, the airway epithelium utilizes a variety of receptors, including TLRs, to detect antigens and infectious microorganisms. The lung and respiratory tract are particularly susceptible to pathogens and allergens because of the constant exposure of inhaled air. Additionally, TLRs recognize exogenous pathogen-associated molecular patterns (PAMPs) and host-derived damage-associated molecular patterns (DAMPs; Lafferty et al., 2010). The activation of TLRs through these means selectively induce inflammation, inflammatory cell recruitment, and cytokine release. In particular, TLR2 and TLR4 are regarded as the major TLRs responsible for sustaining the inflammatory responses in both asthma and COPD. However, altering the immunoreactivity and expression of TLRs may result in an impaired immune response, which is marked by abnormal inflammation in the aforementioned diseases (Lafferty et al., 2010). This review will highlight the roles of TLRs in inflammation and their association to the physiopathology of asthma and COPD, as well as discuss the potential for TLR-based treatment of these diseases.

## Molecular characteristics of asthma and COPD

Persistent inflammation and airflow obstruction are the major characteristics of asthma and COPD. However, the patterns of inflammation and the immunological mechanisms that lead to the airway structural alterations are different for the respective diseases (Barnes, 2000, 2008). Inflammation in the larger conducting airways is mainly observed in asthma. In contrast, COPD predominantly affects the lung parenchyma and smaller airways (Barnes, 2008). Asthma is characterized by the presence of eosinophils, mast cells, and CD4^+^ T lymphocytes; whereas COPD is distinguished by the dominance of neutrophils, macrophages and CD8^+^ T lymphocytes in the respiratory tract (Buist, 2003; Baines et al., 2011; Athanazio, 2012). T cell activation is dependent upon the antigen presenting cells (e.g., dendritic cells) and plays a substantial role in inflammation (Shalaby and Martin, 2010).

**Figure 1:**
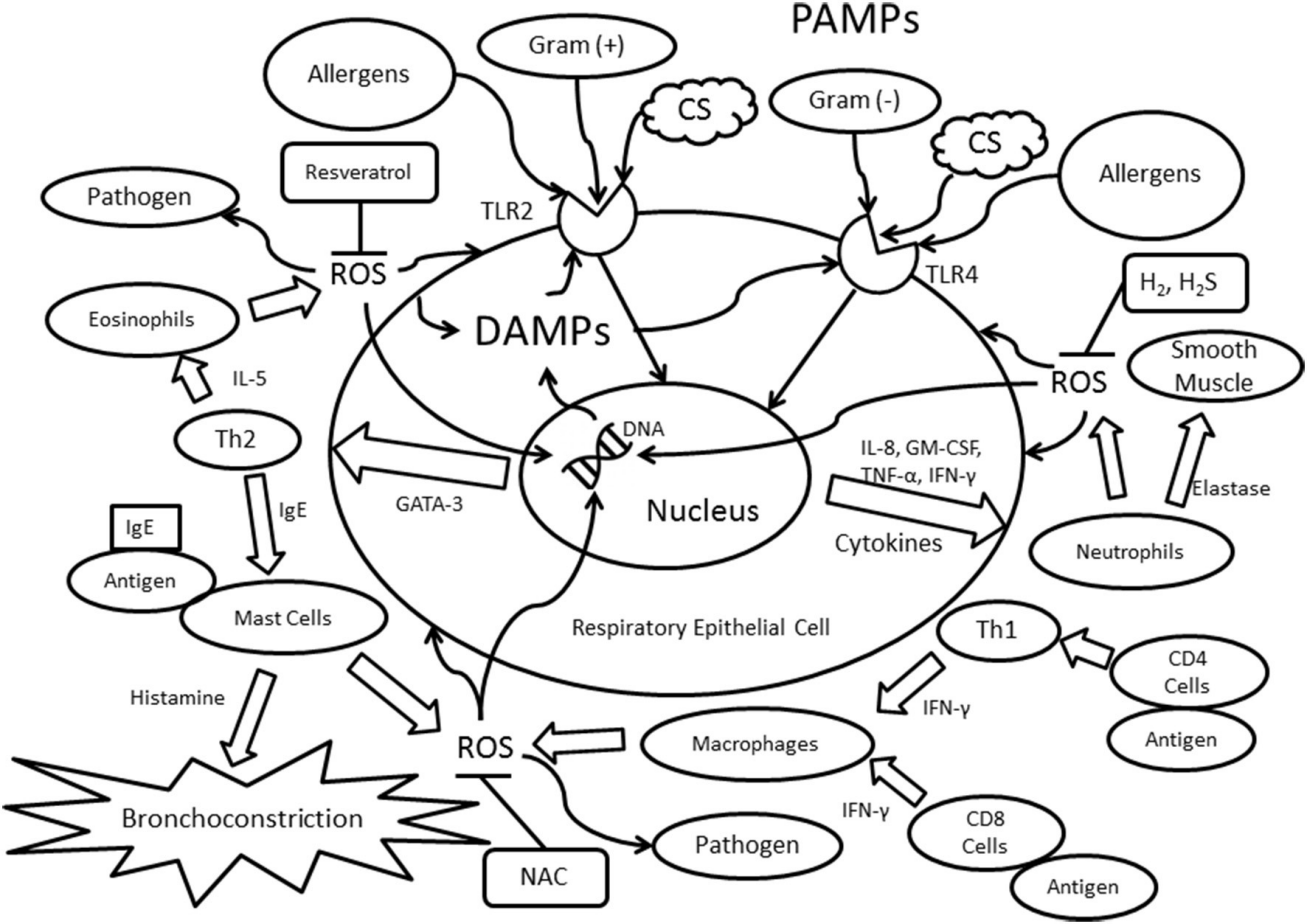
**Immunological mechanisms involving TLR2/TLR4 and Th1/Th2 immunity**. Abbreviations: PAMPs, pathogen-associated molecular patterns; ROS, reactive oxygen species; IL, interleukin; IFN-γ, interferon-γ; TLR, toll-like receptor; CS, cigarette smoke; CD4, cluster of differentiation 4; CD8, cluster of differentiation 8; Th1, T helper cell type 1; Th2, T helper cell type 2; Gram (−), gram-negative bacteria; Gram (+), gram-positive bacteria; IgE, Immunoglobulin E; GATA-3, GATA binding protein 3; TNF-α, tumor necrosis factor-α; GM-CSF, Granulocyte-macrophage colony-stimulating factor; NAC, N-Acetyl-L-cysteine; H_2_, molecular hydrogen; H_2_S, dihydrogen sulfide.

In asthma, IgE binds to receptors found on the surface of mast cells, thus initiating an allergic response (Gauvreau et al., 2015). The recruited mast cells subsequently degranulate to release histamine, an organic compound known to increase hypersensitivity and inflammation, thereby leading to the development of bronchoconstriction (Figure 1; Jutel et al., 2009). Bronchoconstriction is both spontaneous and reversible in asthmatics, and is induced by the hyperreactive smooth muscle surrounding the bronchioles (Athanazio, 2012). Moreover, structural changes, such as subepithelial fibrosis and smooth muscle cell hyperplasia and hypertrophy, can contribute to the thickening of asthmatic airways (Barnes, 2008). In most asthma cases, there is a predominant expression of Th2-type cytokines, including interleukin (IL)-4, IL-5, and IL-13, which are all activated by the transcription factor GATA-binding protein 3 (GATA3; Zhu et al., 2006). These Th2-type cytokines result in the increased migration of eosinophils and mast cells. One of the consequences of eosinophilic inflammation is epithelial shedding, which is frequently observed in biopsies from asthmatic patients (Barnes, 2000).

Interestingly, the activation of mast cells is not evident in patients with COPD (Barnes, 2008). Instead, macrophages are recruited to the COPD airway via the release of the cytokine interferon γ (IFN-γ) by either CD4^+^ T cells or Th1 cells (Barnes, 2004). The reduction in airway capacity originates from cellular damage found in COPD. Several initiators can cause this destruction, including exposure to noxious particles, such as cigarette smoke and exogenous reactive oxygen species (ROS; Zuo et al., 2014). In addition to IFN-γ, other major cytokines expressed in COPD are IL-1, IL-8 (also known as CXCL8), leukotriene B4, granulocyte-macrophage colony-stimulating factor (GM-CSF), and tumor necrosis factor-α (TNF-α) (Kim and Rhee, 2010; Athanazio, 2012). The expression of IFN-γ, IL-8, GM-CSF, and TNF-α lead to the activation and migration of neutrophils to the airways (Figure 1; Wright et al., 2010). Moreover, COPD differs from asthma in the expression of certain TLRs. For instance, in asthma, the presence of TLR4 is lowered in the respiratory tract, whereas TLR4 expression is elevated in COPD patients, possibly as a result of the cellular breakdown in the airways (Bezemer et al., 2012).

## Role of TLR during inflammation

The epithelium of the respiratory tract contains a myriad of TLRs, which participates in the activation of immune responses. Particularly, the role of specific TLRs during inflammation has suggested their involvement in the pathogenesis of asthma and COPD (Lafferty et al., 2010). TLRs are also expressed in resident lung cells, such as alveolar macrophages, and other infiltrating cells (Lafferty et al., 2010). TLR4-expressing fibroblasts and epithelial cells have been found to contribute to the localized inflammatory response (Buckley, 2011). These cells have an anchor-like function, utilizing their ability to attract and immobilize leukocytes through the release of several chemokines and cellular adhesion molecules (Buckley et al., 2001). When the leukocytes persist in the respiratory tract, chronic inflammation is an expected outcome (Buckley et al., 2001; Buckley, 2011).

### Inflammatory aspects of asthma

TLR2 and TLR4 are the most relevant to the onset of asthma and to the inflammatory responses underlying asthmatic exacerbations. TLR4 detects Gram-negative bacteria through their lipopolysaccharides (LPS; Lafferty et al., 2010), while TLR2 plays a large role in recognizing Gram-positive bacteria (Jiang et al., 2006). As described earlier, Th2 cells, mast cells, and eosinophils are commonly associated with the innate and adaptive immune responses in asthma (Figure 1; Walsh et al., 2010). The recognition of allergens, such as house dust mites (HDM), can activate TLR4 and subsequently allergen-specific Th2 cells (Lafferty et al., 2010). TLR2 promotes Th2-biased immune responses, which may be correlated to the Th1/Th2 imbalance in asthma (Phipps et al., 2007). There are two distinct pathways in TLR signaling: myeloid differentiation factor 88 (MyD88)-dependent and MyD88-independent. Both pathways are crucial in regard to the innate immune response. MyD88 and Toll/IL-1 receptor-domain containing adapter-inducing interferon-β (TRIF) bind independently to TLRs, leading to the release of cytokines such as TNF-α, IL-1β, CXCL10, IL-6, and IFN-γ (Piras and Selvarajoo, 2014). During acute asthmatic exacerbations, the cleavage products of proteinases, such as fibrinogen, bind to TLR4s that are present in both the airway epithelium and macrophages, and the binding of these products results in allergic inflammation (Millien et al., 2013). In addition, asthmatic patients who ultimately die have increased expressions of TLR2, TLR3, and TLR4, suggesting their potential role in the development of severe or even fatal asthmatic exacerbations (Kim and Rhee, 2010; Ferreira et al., 2012).

### Genetic factors in asthma

Genetic polymorphisms play a significant role in how individuals respond to diseases. The genetic make-up of a person can make them more or less susceptible to a specific disease (Lee et al., 2012). Similarly, genetic polymorphisms affect the susceptibility, severity, and responsiveness of asthmatic patients to specific allergens. Variants of the TLR4 gene may either increase or decrease the sensitivity of the receptor to allergens (Cho et al., 2011). Zhang et al. indicated that polymorphisms in TLR4 have an impact on the responsiveness of the receptor to pathogens (Zhang et al., 2011). During this study, several types of polymorphisms were examined in an attempt to discover the particular combination of genes responsible for the susceptibility and severity of asthma. The TT homozygote allele of the TLR gene rs1927914 has deleterious effects for the forced expiratory volume in 1 s (FEV_1_) (Zhang et al., 2011). A decreased FEV_1_ suggests a reduced lung capacity and increased airway resistance. Furthermore, the C allele of rs1927914 and the A allele of both rs10983755 and rs1927907 lessen the severity of asthma (Zhang et al., 2011). In addition to TLR4, polymorphisms present in the TLR2 gene are also associated with the risk of asthma development and overall lung function (Gao et al., 2013). The genotype of the TT variant of the homozygous polymorphism rs7656411 in TLR2 may reduce the incidence of asthma (Qian et al., 2010). It has been shown that the T allele of the rs2381289 single nucleotide polymorphism (SNP) in TLR6 contributes to the development of allergic rhinitis and asthma, while the A allele of rs11466651 SNP in TLR10 is negatively associated with asthma development (Qian et al., 2010).

### Inflammatory aspects of COPD

Several TLRs, including TLR2, TLR4, and TLR9, participate in the pathogenesis of COPD. The major risk factors associated with COPD development include cigarette smoking and the inhalation of air pollutants (Bezemer et al., 2012). There is a high correlation between cigarette smoke exposure and the increased gene expression of TLR4 and TLR9 as well as cytokine overproduction (Nadigel et al., 2011; Bezemer et al., 2012; Freeman et al., 2013). Both TLR4 and TLR9 have been shown to contribute to the release of IL-8 from CD8^+^ T cells (Nadigel et al., 2011). Through the upregulation of IL-8, TLR9 elicits an inflammatory response by initiating neutrophil recruitment (József et al., 2006; Mortaz et al., 2010). It is also known that TLR4 deficiency promotes emphysema (An et al., 2012). When exposed to cigarette smoke, alveolar apoptosis increased in TLR4-deficient mice, suggesting a protective role of TLR4 in cell regulation and apoptosis prevention (An et al., 2012). Interestingly, mice that lack TLR4 expression are more susceptible to oxidative stress due to an upregulation of NADPH oxidase (Nox) 3 (Zhang et al., 2006; Kampfrath et al., 2011). It was proposed by Zhang et al. that TLR4 acts as a suppressor of Nox3, therefore regulating the release of ROS (Zhang et al., 2006). Thus, it is possible that in the absence of TLR4, ROS reaches harmful levels and ultimately leads to cellular apoptosis and emphysema. Experimentally, emphysema progression in TLR4-deficient mice can be halted by a Nox inhibitor or Nox3 siRNA (Zhang et al., 2006). Furthermore, a dramatic decrease in antioxidant activity, particularly glutathione levels, was observed in TLR4 knockout mice (Zhang et al., 2006).

Additionally, TLRs are considerably involved with exacerbations of COPD. Research has indicated that MyD88 works in conjunction with TLR4 to upregulate IL kinases and increase IL production (Doz et al., 2008). The interactions between MyD88, TLR4 and IL-1 receptor type I (IL1R1) can lead to acute lung inflammation in COPD patients. Moreover, neutrophil recruitment due to cigarette smoke exposure is dependent on TLR4/MyD88/IL1R1 signaling (Doz et al., 2008; Sarir et al., 2008). TLR2 and TLR4 are the primary mediators that elicit immune responses to microbial invasion in the respiratory tract (Figure 1; Oliveira-Nascimento et al., 2012). Patients with COPD have increased TLR1 and TLR2 expression on CD8^+^ T cells and which may contribute to lung damage and alveolar destruction (Freeman et al., 2013). However, it was also discovered that LPS-induced TLR4 expression is reduced in the lymphocytes of smokers with and without COPD (Knobloch et al., 2011). Therefore, a thorough examination of immune cells and the expression of specific TLRs are needed in future research.

### Genetic factors in COPD

Several polymorphisms are known to influence the likelihood of developing COPD. In particular, SNPs have been shown to impair TLR signaling by reducing the responsiveness of TLRs and the resistance to bacteria and viruses (Cheng et al., 2007; Bronkhorst et al., 2013). Previous research has indicated that the presence of the TLR4-T399I polymorphism increases the risk for COPD development by a factor of 2.4 (Speletas et al., 2009). Several SNPs present in TLR2 are highly associated with lung function. Accordingly, Budulac et al. suggested that both TLR2 SNPs (rs1898830 and rs11938228) are involved in FEV_1_ decline, whereas the SNPs rs7656411 and rs4696480 are related to increased FEV_1_ (Budulac et al., 2012). The TLR2 SNP rs11938228 may be responsible for an acute increase in neutrophils and macrophages. Likewise, the SNPs rs12377632 and rs10759931 in TLR4 are highly associated with inflammation in the lung and respiratory tracts (Budulac et al., 2012). These studies confirm the association between specific polymorphisms and COPD, warranting additional investigations of this area.

## Role of ROS in TLR regulation in asthma and COPD

The accumulation of ROS is also evident in patients with COPD and asthma (Zuo et al., 2012, 2013). ROS are beneficial during immune responses, as their presence aids in the destruction of microbes. However, excessive ROS can directly initiate inflammatory responses and negatively affect tissue functions and cellular structures, including DNA and lipids (Figure 1; Kirkham and Rahman, 2006; Zuo et al., 2011a,b, 2015). These oxidants are produced endogenously by mitochondria, NADPH oxidase, phagocytes, and lymphocytes, or exogenously from sources such as cigarette smoke and ozone (Zuo et al., 2011c; Hernandez et al., 2012). The overproduction of ROS may lead to inflammation via the activation of transcription factors such as nuclear factor kappa-light-chain-enhancer of activated B cells (NF-κB; Rahman, 2006). In addition, ROS can alter the conformation of proteins, which then may bind to antibodies and generate a false immune response, promoting smooth muscle cell hyperactivity via calcium influx (Tetley, 2005; Kirkham et al., 2011). ROS also stimulate TLR2 and TLR4 through MyD88-dependent pathways, inducing cellular damage and elevated levels of DAMPs (Hansel and Barnes, 2009). Furthermore, oxidative stress contributes to the sensitization of allergens by generating an enhanced allergic immune response (Shalaby et al., 2013), thus exacerbating the development of allergic asthma. The presence of ROS in the innate immune response is intended to destroy invading pathogens, yet it may generate undesired cellular damage (Figure 1; Quinn and Schepetkin, 2009; Delfino et al., 2011).

## TLR-based therapy for asthma and COPD

As reported by the Mayo Clinic, the typical treatment for asthma exacerbations includes the inhalation of corticosteroids (Townley and Suliaman, 1987). In the treatment of COPD, a combination of long acting bronchodilators and inhaled corticosteroids is typically used (Wedzicha et al., 2012). Corticosteroids are known to enhance β-adrenergic responses and repress inflammatory responses in the airway (Tamm et al., 2012). However, in severe cases of asthma and COPD, corticosteroids are ineffective in alleviating symptoms (Kirkham and Rahman, 2006; Brusselle et al., 2011; Barnes, 2013). This is likely due to overwhelming oxidative stress and subsequent DNA damage, leading to decreased activity of transcriptional co-repressors such as histone deacetylase-2 (HDAC-2) (Kirkham and Rahman, 2006). Therefore, antioxidant treatments may reduce the production of DAMPs by scavenging ROS and consequently inhibiting the activation of more TLRs (Kirkham and Rahman, 2006).

Drugs that target TLRs can be classified as either agonists or antagonists. TLR agonists increase the response of receptors, while antagonists generally mitigate the responses by attenuating inflammation (Xiang et al., 2010). In allergic asthma, chronic inflammation is caused by exposure to allergens. In asthma treatment, antagonists targeting muscarinic receptors are used to relax the smooth muscle of the airway to promote bronchodilation (Barnes, 2006; Moulton and Fryer, 2011). Shalaby et al. observed that an intranasal administration of Protollin, a compound composed of both TLR2 and TLR4 ligands, is effective in inhibiting allergic responses (Shalaby et al., 2012). Another study conducted by Xirakia et al. showed that the administration of a compound commonly known as Resiquimod or R-848, a TLR7 agonist, was beneficial in the suppression of allergic airway diseases (Xirakia et al., 2010). TLR7 has anti-inflammatory characteristics, and its activation is known to curb inflammation through the reduction of leukocytes entering the airways. Most notably, R-848 diminishes leukocyte recruitment, as well as the production of IL-5 and IL-13 (Drake et al., 2012). The protective mechanisms of TLRs are thought to arise from the reorientation of the immune system to reduce Th2 function, including the production of Th2 cytokines, eosinophils, and bronchial hypersensitivity, resulting in decreased airway inflammation (Aumeunier et al., 2010; Xirakia et al., 2010).

## Key studies in TLRs—an update from 2010 to 2014

TLRs have become a protein of interest and are accepted as a major site for transmitting inflammatory responses in the mucosal lining of the lungs and nasal pathways (Lafferty et al., 2010). However, the activation of the innate immunity in the nasal and lung mucosa is TLR- and PAMP-specific. Ryu et al. observed that HDM-derived β-glucans trigger innate immune responses in the nasal mucosa of mice via TLR2, whereas LPS induces TLR4 signaling in the mucosa of the lower respiratory region (Ryu et al., 2013). The study also found that dual oxidase 2-generated ROS regulates the activation of the β-glucan-induced TLR2 pathway and the LPS-TLR4 interaction (Ryu et al., 2013). The interaction consists of the Toll/IL1R homology (TIR) domain made of the accessory protein CD14, which binds to LPS, TLR4, and MD2 protein. Once LPS is bound, the TIR domain communicates with MyD88 to further relay several kinase activations, such as IL-1 receptor kinase (IRAK), until a response is triggered (Park and Lee, 2013). Collectively, these findings suggest a promising role for TLRs in the treatment of both allergic rhinitis and asthma.

Lucas et al. suggested that TLRs play a pathogenic role in the development of many human diseases, including cardiovascular disease, Parkinson's disease, and autism (Lucas and Maes, 2013b). Additionally, the study examined several methods that attempt to neutralize the effects of TLRs in disease development (Lucas and Maes, 2013b). Several different PAMPs and DAMPs can be recognized by specific TLRs. For instance, the interaction that occurs between LPS and TLR4 involves the binding of LPS to CD14, an accessory protein of TLR4. LPS is then transferred to myeloid differential protein-2, another TLR4 accessory molecule, initiating the downstream signaling pathway of TLR4 (Lucas and Maes, 2013b). Other factors known to affect the TLR pathways were also discussed, including ozone, particular matter, bacteria and viruses, aerosol particles, certain metals, adjuvants, vaccines, pesticides, preservatives, ionizing radiation, inhaled toluene, TLR adaptations, ROS, and Oxidized 1-palmitoyl-2-arachidonyl-sn-3 (OxPAPC; Lucas and Maes, 2013b). Synthetic anti-LPS peptides (SALPs) have been developed as treatments that target LPS. SALPs bind to LPS and prevent TLR4 activation, thereby attenuating the host inflammatory response (Lucas and Maes, 2013b). These SALPs are useful because they effectively neutralize endotoxins at low concentrations, preventing endotoxic shock. There are several antagonists known to suppress the MyD88-dependent and -independent pathways by inhibiting the TANK-binding kinase 1 (TBK1), including compounds found in common traditional Chinese medicines, such as green tea and ginger (Lucas and Maes, 2013b). Targeting the MyD88 pathway may provide potential treatment options because all TLRs, except TLR3, depend on this pathway. Antioxidant treatments, such as N-acetyl-L-cysteine (NAC) and flavonoids, scavenge excess ROS, further reducing the formation of DAMPs (Lucas and Maes, 2013b). By modulating the responsiveness of TLRs through antioxidants, multiple TLR-dependent human diseases may be significantly improved (Lucas and Maes, 2013b). TLR4 antagonists can target the TLR radical cycle. Interestingly, several plants used in traditional Chinese medicine were used to optimize the TLR radical cycle effects (Table 1).

**Table 1:** **Traditional medicine with pharmacologically active substances and their effects**.

**Plants/Pharmacologically active substances**	**Effects**	**Citations**
Green Tea (*Camellia sinensis*) Epigallocatechin gallate	• Potent antioxidant • Decreases TLR4 activity • Excessive exposure leads to green tea-induced asthma	Shirai et al., 2003; Wu et al., 2012; Bao et al., 2014
Turmeric *(Curcuma longa)* Curcumin	• Prevents accumulation of inflammatory cells in airways • Treatments attenuated in an asthma model the Treg/Th17 balance • Reduces NF-κB translocation in cell culture and mice asthma model	Oh et al., 2011; Ma et al., 2013; Chauhan et al., 2014
True cinnamon (*Cinnamomum verum*) Cinnamaldehyde, cinnamic acid, and cinnamate	• Lowers expression of TLR4, MyD88 and NF-κB • Expresses antimicrobial, antiviral and antioxidant activities	Shen et al., 2012; Lucas and Maes, 2013a
Ginger (*Zingiber officinale*)/6-gingerol, 8-gingerol, 6-shogaol	• Anti-inflammatory by modulation of STAT3 and MAPKs signaling pathways • 6-Shogaol induces AhR regulation through transcription factors and gene expression • Reduces airway hyperresponsiveness, by altering Ca^2+^ regulation, relaxes airway smooth muscle	Townsend et al., 2013; Prasad and Aggarwal, 2014; Yoshida et al., 2014
Xing-ren (*Semen Armeniacae Amarum)/*Amygdalin	• Inhibits expression of TNF-α and IL-1β	Huang et al., 2013
Xinjiang liquorice, *(Glycyrrhiza inflata)*, Flavonoids^†^	• Attenuation of allergic airway inflammation • Inhibition of cytokines, e.g., IL-4, IL-5 and IL-13	Chu et al., 2013; Fu et al., 2013

† Licochalcone A, 5-(1,1-Dimethylallyl)-3,4,4′-trihydroxy-2-methoxychalcone, Licochalcone B, Echinatin, Glycycoumarin, Glyurallin B.

*AhR, aryl hydrocarbon receptor; Ca^2+^, calcium ion; IL, interleukin; MAPK, mitogen-activated protein kinase; MyD88, myeloid differentiation primary response gene (88); STAT3, signal transducer and activator of transcription 3; NF-κB, nuclear factor kappa-light-chain-enhancer of activated B cells; Th17, T helper 17 cell; TLR, toll like receptor; TNF-α, tumor necrosis factor alpha; Treg, regulatory T cell*.

**Figure 2:**
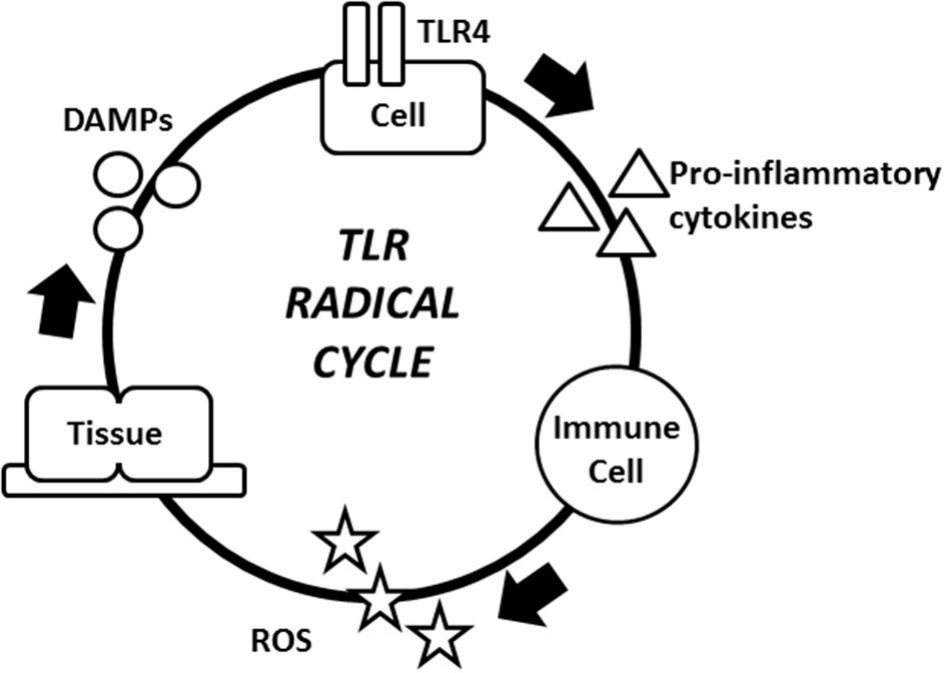
**The TLR radical cycle**. The TLR4 receptor on any TLR4 expressing cell, e.g., an epithelial cell or a fibroblast is triggered by PAMPs and/or DAMPs. These immobile cells are part of the inflammatory cycle and they regulate the location of the inflammation. After TLR4 is activated, pro-inflammatory cytokines are released and started to attract macrophages, mast cells and neutrophils, ultimately leading to ROS production. ROS attack microbes, but also cause collateral damage to the cell. Endogenous molecules are modified by ROS and act as DAMPs to reactivate TLR4. The loop is then complete and another cycle may begin. The result of the cycle is the persistence of chronic inflammation. Abbreviations: DAMPs, damage-associated molecular patterns; PAMPs, pathogen-associated molecular patterns; ROS, reactive oxygen species; TLR4, toll-like receptor 4.

TLR activation, pro-inflammatory cytokine production, ROS release, and the production of DAMPs that reactivate TLRs all comprise the TLR radical cycle (Figure 2). Particularly, TLR4 activation results in the translocation of transcription factors, such as NF-κB and activator protein 1 (AP1), from the cytoplasm into the nucleus, which in turn initiates the transcription of pro-inflammatory cytokines, such as TNF-α, IL-1, IL-6, and IL-8 (Lucas and Maes, 2013b). These pro-inflammatory cytokines and similar compounds, such as extracellular RNA, can attract different leukocytes to release ROS (Fischer et al., 2012). Typical types of ROS, such as hypochlorite (OCl^−^), hydrogen peroxide (H_2_O_2_), singlet oxygen (O_2_(a^1^Δ_*g*_)), hydroxyl radical (^∙^OH) and superoxide (${\text{O}}_{2}{}^{•-}$), can destroy bacteria and viruses and degrade lipids, proteins, and sugars (Uy et al., 2011; Bachi et al., 2013; Nyström et al., 2013). Some of the oxidized compounds that result from exposure to ROS include oxidized phospholipids and degraded hyaluronan fragments (Kadl et al., 2011; Muto et al., 2014). These altered molecules act as DAMPs, and each has the ability to activate TLRs, most notably TLR4 (Figure 2; Lucas and Maes, 2013b). This activation of TLR4 by DAMPs is the final stage of the cycle. DAMPs then “reinitiate” a new round of the cycle consisting of PRRs, pro-inflammatory cytokines, ROS, and DAMPs (Lucas and Maes, 2013b). This cycle becomes independent of its initial trigger and establishes a dramatic amplification of the immune response (Lucas and Maes, 2013b). The ongoing cycle is controlled by multiple factors, and there is no clear initiation or termination. The cycle provides a multitude of targets ranging from TLRs to oxidative radicals for suppressing the immune response. Further research is needed to determine which of these could translate into effective treatment approaches.

TLR5 and TLR7 are epithelial receptors that have both been shown to participate in respiratory inflammation (Shikhagaie et al., 2014). Specifically, TLR5 aids in the detection of bacterial flagellin protein, a component of flagellated bacteria, and enhances flagellin-specific CD4^+^ T cell responses and adaptive immunity (Letran et al., 2011). In addition, TLR7 represses host inflammatory responses by promoting bronchodilation, as well as decreasing airway hyperreactivity and inflammation (Drake et al., 2012). The expressions of TLR5 and TLR7 are decreased in the epithelium of patients with severe asthma compared with healthy individuals and those with moderate asthma. This decreased expression may affect the innate immune response (Shikhagaie et al., 2014). Metcalfe et al. studied the effects of several PAMPs on TLR expression in alveolar macrophages in patients with COPD, such as cigarette smoke extract (CSE), common LPS, Pam3CSK4, and phase 1 flagellin (FliC) (Metcalfe et al., 2014). The study found that CSE suppressed the TLR-induced production of TNF-α, IL-6, and IL-10. However, IL-8 production was unaffected. An analysis of gene expression illustrated that CSE inhibited LPS-activated TNF-α transcription but did not affect the transcription of IL-8 in alveolar macrophages. This attenuating effect has been correlated to a decreased activation of p38, p65, and extracellular signal regulated kinase (ERK) (Metcalfe et al., 2014).

## Potential therapeutics targeting inflammation and ROS

Considering ROS formation is common in asthma and COPD, the use of free radical scavengers, such as resveratrol, NAC, hydrogen sulfide (H_2_S), and molecular hydrogen (H_2_), may be an effective adjuvant treatment (Kirkham and Rahman, 2006; Ohta, 2011; Wang et al., 2012; Jiang et al., 2014). For instance, airway hyperreactivity can be alleviated by NAC treatment (Carlsten et al., 2014). In an animal model of allergic asthma, the administration of NAC attenuated airway hyperresponsiveness induced by birch pollens (Shalaby et al., 2013). In addition, immune responses initiated by diesel exhaust exposure can be diminished via NAC supplementation (Carlsten et al., 2014). In an animal model for allergic asthma, resveratrol, a well-known flavonoid, restored inositol polyphosphate 4 phosphatase (INPP4A) activity and thus reduced allergic reactions (Aich et al., 2012). This compound interacts with the aryl hydrocarbon receptor (AhR), which can downregulate the expression of pro-inflammatory genes (Revel et al., 2003). H_2_S is another plausible target for the treatment of asthma because it may serve as a molecular marker for asthma, it could be used as a therapeutic gas, and it may act as both a hormone and a neurotransmitter in the human body (Farrugia and Szurszewski, 2014; Lo Faro et al., 2014). H_2_S can relax the smooth muscle in intrapulmonary airways by inhibiting intracellular Ca^2+^ release (Castro-Piedras and Perez-Zoghbi, 2013). On the other hand, H_2_S exhibits antioxidant and anti-inflammatory characteristics (Zhang et al., 2013). For example, exogenous H_2_S can reverse ovalbumin-induced asthma (Zhang et al., 2013). The concentration of H_2_S is increased in the sputum supernatants and the serum of asthma patients, while lung function is found to be inversely correlated with H_2_S levels in sputum (Chung, 2014). Therefore, it is possible that H_2_S may serve as a molecular marker in patients diagnosed with asthma (Chung, 2014).

Interestingly, H_2_ appears to be another effective ROS scavenging drug (Ohno et al., 2012). In a rat model of COPD, the beneficial effects of the injection of H_2_-enriched water included reduced mucus production and epithelium damage, partly associated with the scavenging of free radicals by H_2_ (Ning et al., 2013). The positive effects of H_2_-enriched saline have been reproduced in animal models of asthma (Xiao et al., 2013). Xiao et al. demonstrated that the application of saline enriched with H_2_ can attenuate the NFκB pathway, an important part of the TLR radical cycle (Xiao et al., 2013). Thus, the inhalation of gaseous H_2_ may be a viable treatment option for patients with asthma and/or COPD.

## Conclusion

Current research has shown the significance of TLRs in the pathogenesis of respiratory diseases, such as asthma and COPD. The role of TLRs in the immune response has been well-documented, and polymorphisms of the TLR genes can result in drastic changes in the severity and susceptibility of respiratory inflammatory diseases. Several treatments targeting the TLR pathway have been topics of ongoing research in an attempt to minimize the severity of both diseases. Studies relating to asthma mostly show a higher degree of activation of the TLR radical cycle. In contrast, this cycle seems to collapse in COPD subjects. Thus, antagonists of TLRs and anti-inflammatory drugs may have beneficial effects in patients with asthma but not in those with COPD. However, antioxidant therapies may be beneficial in both diseases. Further research is necessary to explore the realm of TLR-based treatments, as these molecules play a significant role in the development and ongoing symptoms associated with asthma and COPD.

### Conflict of interest statement

Dr. Kurt Lucas is an inventor of the compositions for the preparation of hydrogen enriched water in the international patent application WO2014048953 (A1). The owner of this application is Max Planck Society. All other authors declare that the research was conducted in the absence of any commercial or financial relationships that could be construed as a potential conflict of interest.
